# SARS-CoV-2 spread to endocrine organs is associated with obesity: an autopsy study of COVID-19 cases

**DOI:** 10.1007/s12020-023-03518-0

**Published:** 2023-09-12

**Authors:** Anello Marcello Poma, Alessio Basolo, Greta Alì, Diana Bonuccelli, Iosè Di Stefano, Marco Conti, Paola Mazzetti, Rebecca Sparavelli, Paola Vignali, Elisabetta Macerola, Mauro Pistello, Ferruccio Santini, Fulvio Basolo, Antonio Toniolo

**Affiliations:** 1 Department of Surgical, Medical, Molecular Pathology and Critical Area, University of Pisa, Pisa, Italy; 2 Obesity and Lipodystrophy Center, Endocrinology Unit, University Hospital of Pisa, Pisa, Italy; 3 Department of Forensic Medicine, Azienda USL Toscana Nordovest, Lucca, Italy; 4 Retrovirus Center and Virology Section, Department of Translational Research and New Technologies in Medicine and Surgery, University of Pisa, Pisa, Italy; 5 Global Virus Network, University of Insubria, Varese, Italy

**Keywords:** COVID-19, SARS-CoV-2, Endocrine, Obesity

## Abstract

**Purpose:**

SARS-CoV-2 infection may be limited to the respiratory tract or may spread to multiple organs. Besides disease severity, factors associated with virus spread within the host are elusive. Here, we tried to identify features associated with SARS-CoV-2 spread to endocrine organs.

**Methods:**

In a retrospective autoptic cohort of 51 subjects who died because of COVID-19, we analyzed the severity and type of lung pathology, patients’ features and the detection of virus in thyroid, testis, adrenal gland, pancreas, anterior pituitary, and the white adipose tissue (WAT).

**Results:**

The SARS-CoV-2 genome was detected in endocrine organs of 30/51 cases. The anterior pituitary and WAT were most frequently positive for virus. While pathological features of lung were not associated with the presence of virus in endocrine organs, obesity (BMI > 30) was significantly associated to virus detection in pancreas (*p* = 0.01) and thyroid (*p* = 0.04). WAT infection was detected more frequently in males (*p* = 0.03).

**Conclusion:**

In subject with obesity dying of COVID-19, the virus frequently spreads to endocrine organs. The findings emphasize the need for optimal treatment of patients with obesity at the very onset of COVID-19. Since post-COVID conditions remain a major issue worldwide, a rigorous follow-up of endocrine function—especially of thyroid and pancreas—is advocated in subjects with obesity.

## Introduction

Airborne transmission of the severe acute respiratory syndrome coronavirus 2 (SARS-CoV-2) may result in the clinical onset of COVID-19 that comprises severe lung injury and impaired gas exchange. Pathologic changes include diffuse alveolar damage and infiltration of neutrophils and macrophages, hyaline membranes, vascular changes and micro- or macro-coagulopathies [1, 2]. In later phases of the pandemic, this acute pneumonia scenario is becoming less common due to widespread vaccination, the rise of more transmissible but less pathogenic virus variants, and improved therapeutics [3, 4].

Upon infection, a first line of defense is represented by type I and type III interferons (IFN) that can be generated by most cell types [5]. In low-dose infections, the IFN response usually limits viral replication resulting in moderate damage and mild symptoms. However, in the presence of inborn or acquired IFN defects [6], the replication of SARS-CoV-2 is unrestricted, and the virus may spread to internal organs [7].

In COVID-19, the determinants of disease severity and extrapulmonary complications (EPCs) are incompletely understood. Viremia, however, may predict the severity of COVID-19 and EPCs [8, 9], i.e., the possible spread of virus to multiple tissues, including vascular endothelia [10, 11], heart [12], kidneys [13], brain [14], and the endocrine system [15]. Some of these targets may act as virus reservoirs that consent the protracted replication of SARS-CoV-2 [16–18].

In subjects deceased of COVID-19, we substantiated the presence of SARS-CoV-2 in thyroid, testis, adrenal gland, pancreas, anterior pituitary, and the white adipose tissue (WAT) [19–24]. Infection of each of these organs is associated with activation of a type I IFN response and with deregulation of endocrine-specific gene transcripts [22–24] that may contribute to endocrine dysfunctions in COVID-19 survivors [25–27].

Recognizing the factors associated with the extrapulmonary dissemination of SARS-CoV-2 will provide leads to design ways for limiting systemic infection and the spread of virus to internal organs. For instance, the early administration of IFN lambda has been demonstrated to prevent severe forms of the disease [28].

In subjects who died of COVID-19 we attempted to correlate both the severity and type of lung pathology as well as patients’ features with the presence of virus into endocrine organs. Results indicate that obesity is an important factor associated with the dissemination of SARS-CoV-2 to the endocrine system.

## Materials and methods

### Study cohort and pathological examination

This is a retrospective observational study of 51 autopsy cases from individuals who died because of COVID-19 as defined by WHO guidelines [29]. Cases were collected between November 2020 and December 2021. The study was conducted in accordance with the Declaration of Helsinki with later amendments and was approved by the local Ethics Committee (Comitato Etico Area Vasta Nord-Ovest, Italy; protocol number 17327; May 14, 2020). Some of the investigated cases overlap with those of published studies [19–24].

In all cases, a pre-mortem nasopharyngeal swab and a post-mortem lung specimen tested positive for SARS-CoV-2 genome. Peripheral and central areas of the two lungs were sampled to assess virus positivity at the time of death. Tissues were fixed in 10% formalin and embedded in paraffin. Hematoxylin and eosin stained sections were examined by two pathologists (GA and IDS). The extension of pathologic alterations in lung was evaluated as the percentage of specimens showing changes proper of COVID-19 [30]. The histologic features of lung tissue were evaluated and graded as previously reported [30]: 0, absent; ≤50%, focal; >50%, diffuse. Large vessel thrombi were defined as those detected in arteries/veins over 1.0 mm in diameter. The term microthrombi refer to clots formed in small pulmonary vessels (≤1.0 mm). From the 51 autopsy cases, 172 specimens of the following endocrine organs were tested for the SARS-CoV-2 genome: adrenal gland (*n* = 26), ovary (*n* = 8), pancreas (*n* = 24), pituitary gland (*n* = 28), testicles (*n* = 23), thyroid (*n* = 29), abdominal subcutaneous WAT (*n* = 34).

### RT-PCR assay

RNA was isolated from two to four 10-μm-thick sections of formalin-fixed paraffin-embedded (FFPE) tissue using the RNeasy FFPE kit (Qiagen, Hilden, Germany). RNA quality was assessed by spectrophotometry (Trinean, Gentbrugge, Belgium). About 100 ng of total RNA was tested for SARS-CoV-2 genome using the SARS-CoV-2 WE RT-PCR kit (Diatech Pharmacogenetics, Jesi, Italy). This assay targets two viral genes: the nucleocapsid (N) and the RNA-dependent RNA polymerase (RdRp). As indicated by the manufacturer, cut-off values for considering a sample virus-positive are the 36th cycle threshold (Ct) for the N gene and the 38th Ct for the RdRp gene. The assay has a limit of detection of 5 target copies per reaction. Amplification of at least one of the two viral genes was sufficient to define virus positivity.

### Typing of SARS-CoV-2 variants

Major SARS-CoV-2 variants were assessed using commercial kits based on multiplex real-time RT-PCRs: the Allplex™ SARS-CoV-2 Variants I and II Assays (Seegene, Seoul, Republic of Korea). When used together, the two assays allow identifying the following SARS-CoV-2 variants: original Wuhan strain, Alpha [B.1.1.7], Beta [B.1.351], Gamma [P.1], Delta [B.1.617], and Omicron [B.1.1.529] variants. The PCR typing method uses specific primers and probes to detect mutations in the receptor-binding domain of the SARS-CoV-2 spike protein: ΔHV 69/70, K417T, K417N, L452R, E484K, E484Q, N501Y, P681H, and P681R. The assay’s limit of detection is 100 target copies per reaction. Molecular assays were prepared using the NIMBUS or STARLET IVD Launcher OneStep program (Seegene). Five microliters of each of the following reagents was added to the reaction mixture: buffer, primers and probes, enzymes, sample RNA. Plates were run on a real-time PCR system (CFX96™ IVD, BioRad, Hercules, USA) using the recommended protocol. PCR results were interpreted via the proprietary Seegene software.

### Statistics

Continuous variables are presented as median and interquartile range (IQR). Differences were tested by the Mann–Whitney *U* test. Time-to-event data were tested by log-rank test. For contingency tables, the Fisher’s exact test was used. Concordance among tissues positive for SARS-CoV-2 was estimated using the Cohen’s kappa (*k*) coefficient. Tests were two-tailed and significance level was set at *p* = 0.05. Statistics and graphics were produced in the R environment (v.4.2.2, https://www.r-project.org/, last accessed May 4, 2023).

## Results

### Relationship of lung pathology and virus spread to endocrine organs

The SARS-CoV-2 genome was tested in 2–6 specimens of endocrine organs per case (mean 3.4). SARS-CoV-2 was considered as not spreading to endocrine organs when a minimum of two different tissue types were tested and no positive results were obtained.

In 30 of 51 cases (59%), the SARS-CoV-2 genome was detected in at least one endocrine organ. Cases were stratified according to the presence or absence of SARS-CoV-2 in the endocrine system. The statistical association of virus positivity in endocrine organs with the extent and the histologic features of lung pathology was then evaluated.

**Fig. 1:**
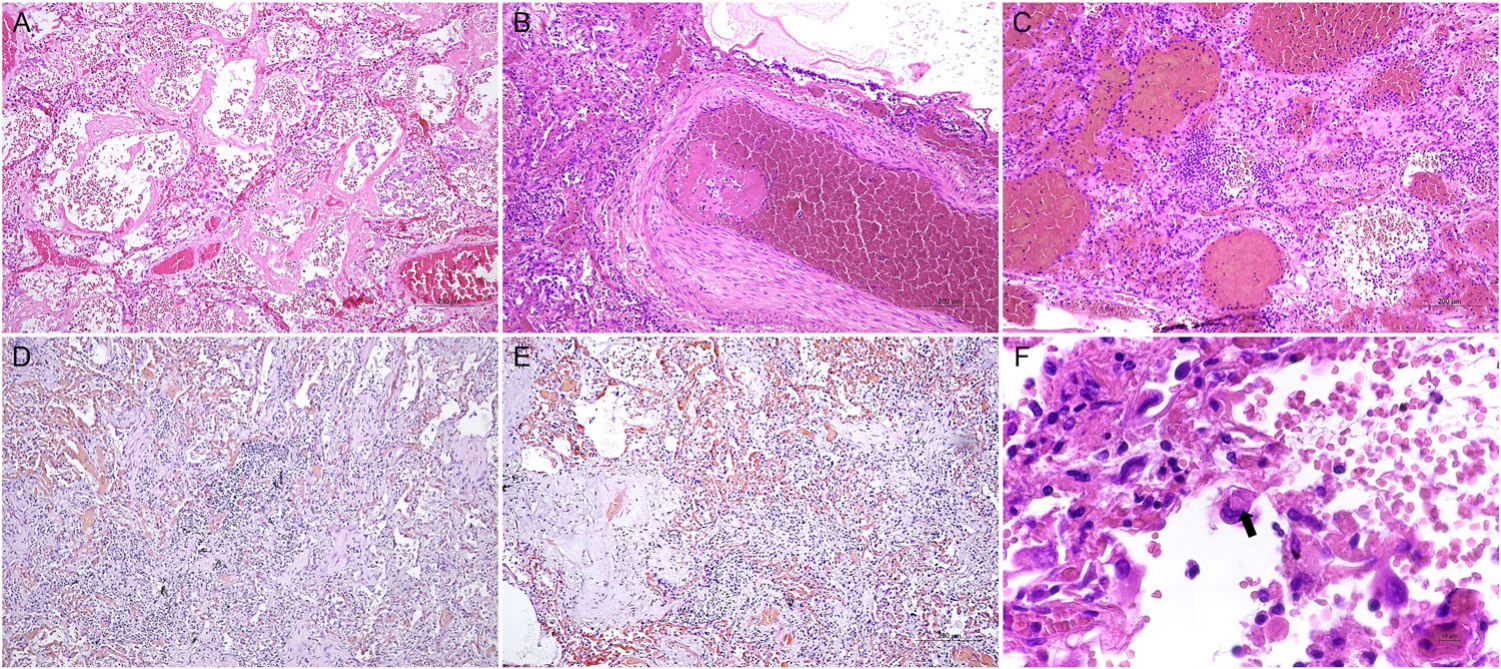
Histologic features of lung parenchyma in severe COVID-19. Hematoxylin and eosin staining: **A** a case with hyaline membranes and type 2 cell hyperplasia (original magnification ×10); **B** fibrin thrombus in an arteriole (×10); **C** acute inflammation of lung parenchyma characterized by neutrophil infiltration and alveolar wall injury (×10); **D** lymphocytic infiltration in chronic inflammation (×10); **E** young collagen with features of organizing pneumonia (×10); **F** high power magnification of an enlarged pneumocyte with intracytoplasmic inclusions (black arrow, ×60)

Table 1 summarizes the extent and the features of lung pathology in the investigated cases. The extension of lung pathology ranged from 50 to 100% but was not significantly related to the presence or absence of virus in endocrine organs (88% in cases with virus in endocrine organs vs. 82% in virus-negative cases on average; *p* = 0.28). Figure 1 shows representative features of lung pathology. Focal to diffuse chronic inflammation of alveoli was found in all cases, acute alveolar inflammation in 46/51 cases (90%), organizing pneumonia in 43/51 cases (86%). Vascular microthrombi were detected in 92% of cases. Thrombi in large vessels were present in about 60% of cases. In some cases, hyaline membranes, intracytoplasmic inclusions, squamous metaplasia, or secondary lesions were also detected.

**Table 1:** Pathological examination of lung tissues from subjects who died of COVID-19

Pathological feature	SARS-CoV-2 detected only in lung (*n* = 21)	SARS-CoV-2 detected also in endocrine organs (*n* = 30)	*p* value
Acute alveolar inflammation, *n* (%)			0.78
Focal	8 (38%)	15 (50%)	
Diffuse	11 (52%)	12 (40%)	
Chronic alveolar inflammation, *n* (%)			0.08
Focal	4 (19%)	13 (43%)	
Diffuse	17 (81%)	17 (57%)	
Hyaline membrane, *n* (%)			0.55
Focal	11 (52%)	11 (37%)	
Diffuse	8 (38%)	16 (53%)	
Pneumocyte type I hyperplasia, *n* (%)			0.34
Focal	10 (47.5%)	9 (30%)	
Diffuse	10 (47.5%)	17 (57%)	
Organizing pneumonia, *n* (%)			0.48
Focal	8 (38%)	17 (57%)	
Diffuse	10 (48%)	10 (33%)	
Alveolar wall acute inflammation, *n* (%)			0.87
Focal	6 (29%)	11 (37%)	
Diffuse	11 (52%)	13 (43%)	
Alveolar wall chronic inflammation, *n* (%)			1
Focal	8 (38%)	11 (37%)	
Diffuse	13 (62%)	19 (63%)	
Vessels microthrombi, *n* (%)			0.69
Focal	7 (33%)	13 (43%)	
Diffuse	12 (57%)	15 (50%)	
Large vessels thrombi, *n* (%)			0.82
Focal	8 (38%)	13 (43%)	
Diffuse	6 (29%)	6 (20%)	
Vasculitis, *n* (%)			0.56
Present	10 (48%)	11 (37%)	
Capillaritis, *n* (%)			0.07
Present	17 (81%)	16 (53%)	
Squamous metaplasia, *n* (%)			0.44
Present	2 (10%)	6 (20%)	
Intracytoplasmatic inclusion, *n* (%)			0.77
Present	9 (43%)	11 (37%)	
Other lesions, *n* (%)			0.72
Present	5 (24%)	5 (17%)	

No significant differences were observed between cases without or with viral spread to extrapulmonary organs

### Relationship of patients’ parameters with virus spread to endocrine organs

**Fig. 2:**
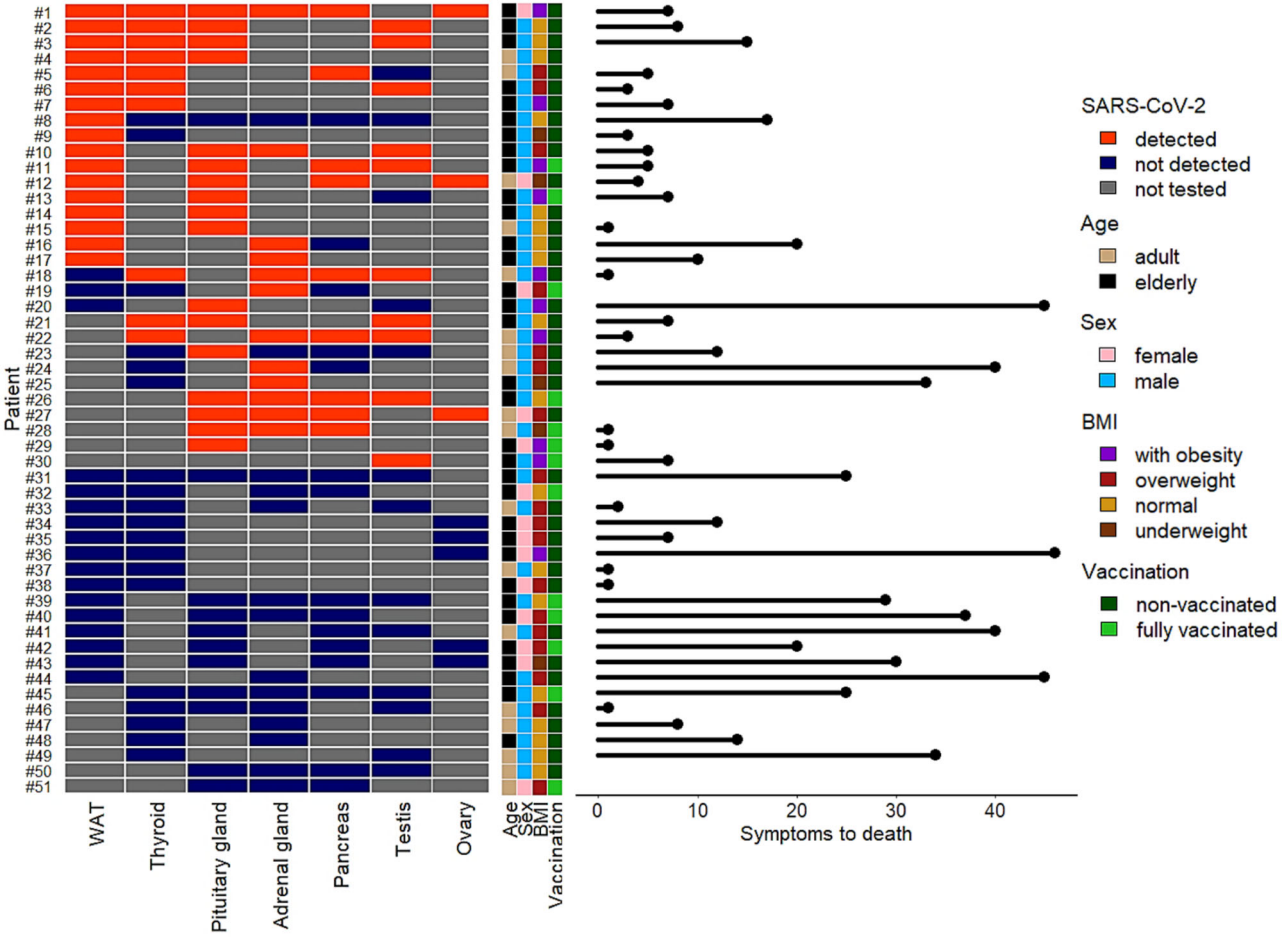
Summary of virus detection and other parameters in endocrine organs. Left side: the heatmap shows detection (orange) or absence (blue) of SARS-CoV-2 genome in the investigated tissues. Not tested tissues are in gray. Each row refers to its case number on the left. Each column represents an endocrine organ. Center: for each case, sidebars show age category (adult or elderly), sex, BMI class, vaccination status. Right side: horizontal lines represent time from the onset of symptoms to death. Blank rows: data not available. Color codes for each parameter are shown on the right

It was then assessed whether patients’ features were associated with the presence of virus in endocrine organs (Fig. 2). As shown in Table 2, both Body Mass Index (BMI) > 30 and short time from the onset of symptoms to death were significantly associated with the presence of virus in the endocrine system (*p* = 0.04 and *p* = 0.05, respectively). In fact, virus spread to endocrine organs was detected in 9/10 cases with BMI > 30, i.e., much more frequently than in subjects with BMI values lower than 30 (*p* = 0.03). In addition, a trend for a positive association was observed for the male sex (*p* = 0.06). Other parameters such as age, comorbidities, use of respiratory supports and time from death to autopsy (a technical parameter that may influence the results of post-mortem assays) were not associated with virus positivity of endocrine organs. Of note, in our cohort no significant differences were observed between subjects not vaccinated against COVID-19 and those that—before contracting COVID-19—had received at least two vaccine doses. However, in Italy, the COVID-19 vaccination started in January 2021, and vaccinated subjects represented a small part of the investigated cases. In fact, the study cohort was collected from November 2020 to December 2021.

**Table 2:** Demographics and clinical features of the study cohort

Features	SARS-CoV-2 detected only in lung (*n* = 21)	SARS-CoV-2 detected also in endocrine organs (*n* = 30)	*p* value
Age, median (IQR)			0.91
Years	68 (55–77)	69 (56–78)	
Sex, *n* (%)			0.06
Male	12 (57%)	25 (83%)	
BMI, *n* (%)			0.04
Underweight	1 (5%)	4 (13%)	
Normal weight	8 (38%)	10 (34%)	
Overweight	11 (52%)	7 (23%)	
With obesity (BMI > 30)	1 (5%)	9 (30%)	
Any comorbidities, *n* (%)			0.77
Yes	14 (67%)	18 (60%)	
Diabetes, *n* (%)			0.49
Yes	3 (14%)	7 (23%)	
Cardiovascular disease, *n* (%)			0.39
Yes	11 (52%)	11 (37%)	
Chronic pulmonary disease, *n* (%)			0.68
Yes	3 (14%)	3 (10%)	
Complete vaccination^a^, *n* (%)			0.75
Yes	6 (29%)	7 (23%)	
Respiratory support, *n* (%)			0.17
Yes	12 (57%)	11 (37%)	
Time from symptoms to death, median (IQR)			0.05
Days	22.5 (7.25–33)	7 (3–12)	
Time from death to autopsy, median (IQR)			0.74
Days	5 (3–5)	5 (2.5–7.5)	

Cases are grouped according to the detection of SARS-CoV-2 in endocrine organs

*IQR* interquartile range, *BMI* body mass index (Kg/m^2^)

^a^One subject had received a single shot of vaccine and was considered not completely vaccinated

The SARS-CoV-2 variants could be established in 18/51 cases. Virus was present in endocrine organs of the 18 cases. The Wuhan variant of SARS-CoV-2 was highly prevalent (14/18 cases), and rare cases were ascribed to the Alpha (2/18) and the Delta (2/18) variants. The Omicron variant was not found in the investigated cohort since, in Italy, it was first detected in November 2021 (i.e., at the end of the study period).

### Endocrine organs infected by SARS-CoV-2 and concordance rate among different organs

**Fig. 3:**
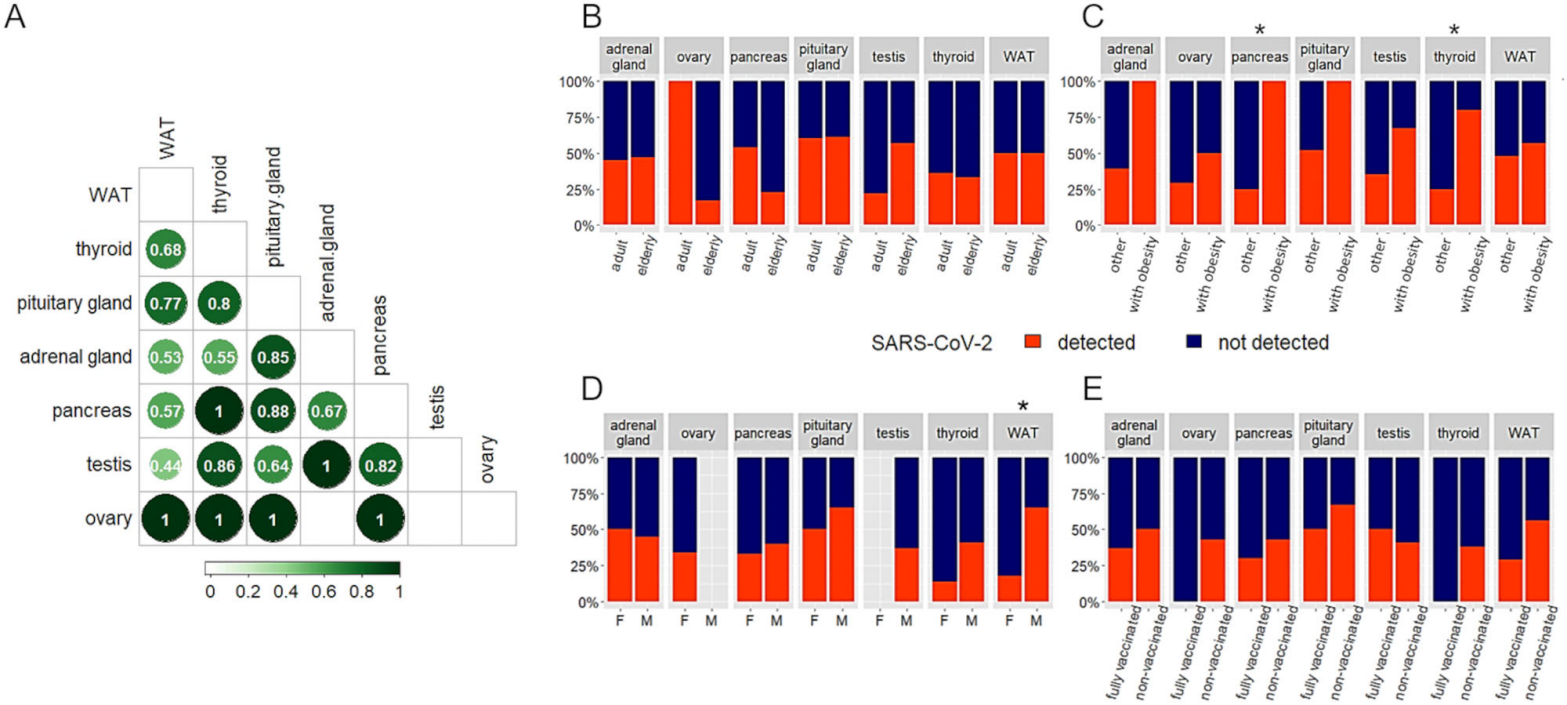
Detection of SARS-CoV-2 in the investigated endocrine organs. **A** Concordance among detection of SARS-CoV-2 genome in endocrine organs was assessed by Cohen’s kappa (*k*) coefficient with 1 denoting perfect concordance. **B** Virus detection in different organs according to age class. **C** Virus detection in subjects without or with obesity. **D** Virus detection according to sex. **E** Virus detection according to vaccination state. Differences were compared using the Fisher’s exact test. Asterisks indicate statistical significance

SARS-CoV-2 was detected in endocrine organs at the following rates: anterior pituitary 17/28 (61%), WAT 17/34 (50%), adrenal gland 12/26 (46%), testicle 10/23 (43%), pancreas 9/24 (37%), ovary 3/8 (37%), thyroid 10/29 (34%). In cases positive for virus in the endocrine system, the concordance among different organs varied extensively. As shown in Fig. 3A, a high concordance (*k* > 0.8) was found among organs with low positive detection rates (i.e., thyroid, pancreas, testis). Ovary had a perfect concordance (*k* = 1) with other endocrine organs, but the number of investigated cases was small (*n* = 8). As shown in Fig. 3, in individuals with BMI values > 30 the virus frequently spread to pancreas (*p* = 0.01) and to the thyroid gland (*p* = 0.04). Infection of WAT was observed more frequently in males than in females (*p* = 0.03). No differences were observed for sex, age, administration of COVID-19 vaccines.

## Discussion

Lungs are the primary target of SARS-CoV-2. Extensive infection of alveoli and pulmonary vasculature may lead to severe hypoxia and death. COVID-19 is heterogeneous in terms of severity and clinical phenotype. The majority of affected patients recover from acute infection, but over 10% continue to suffer from late sequelae [31].

From primary infection targets, viruses can spread to other organs via the bloodstream. Respiratory viruses such as influenza do not typically cause viremia. Conversely, SARS-CoV-2 may enter the blood [32] and the viral load in plasma is predictive of COVID-19 outcomes in hospitalized patients [33]. Endocrine organs are irrorated by capillaries endowed with fenestrae and transendothelial channels. On the outside, pericytes adhere to the basal membrane, regulate blood flow and represent a niche of pluripotent adult stem cells for tissue regeneration [34]. Both endothelial cells and pericytes express the entry factors for SARS-CoV-2 (ACE2, TMPRSS2 and/or vimentin) and may allow virus replication [35–37]. Hence, conditions letting the virus to enter the bloodstream may be essential for its dissemination to the endocrine system.

Here, we compared pathological lung alterations to the spread of SARS-CoV-2 into endocrine organs of subjects who died because of COVID-19. The virus was detected in at least one endocrine organ of about 60% of subjects deceased of COVID-19, but the extension of lung pathology or specific histologic alterations were not associated with the presence of virus in the endocrine system. Autopsies of the present study were performed at a time when aggressive virus variants (Wuhan, Alpha and Delta) were predominant in Italy. Compared to the currently circulating virus strains, the initial SARS-CoV-2 variants were likely more prone to invade the circulatory system and extrapulmonary organs [9]. Virus infection was more frequent in the pituitary gland and WAT, while virus positivity was more rare in testis, ovary, pancreas, and thyroid.

In subjects with obesity, infection of endocrine organs was significantly more common (9/10) than in subjects with BMI value < 30. This observation is of clinical relevance, and no imbalances in terms of age, sex and vaccination state were observed between subjects with and without obesity. The frequent extrapulmonary spread of virus in subjects with adiposity-based chronic disease does not only provide an explanation for adverse outcomes in these individuals [38], but may also represent a reason for the increased risk of long COVID in subjects with obesity [39, 40]. Although in our series only 5 subjects were underweight, the majority of them (i.e., 4) showed SARS-CoV-2 extrapulmonary dissemination. This observation along with the increased risk of severe COVID-19 outcome in underweight people [41] might suggest including these subjects among those requiring prioritized vaccination. Notably, cases in which virus was present in the endocrine system died more rapidly than those in which virus was not detected in endocrine organs. The finding likely points to the association of severe clinical conditions with the spread of virus to extrapulmonary organs.

Finally, this study of severe COVID-19 confirms that endocrine infection is more frequent in males than in females. In fact, it has been demonstrated that sex chromosome dosage does control the antiviral activity of natural killer cells that is less effective in males than in females [42]. Hence, the frequent infection of WAT in males might be a consequence of different sex-related immune efficiency. On the other hand, WAT infection might determine the disproportionate release of pro-inflammatory cytokines, thus playing a role in the worse prognosis.

Limitations of the present study include: (1) the small sample size with low statistical power in organ-specific analyses. Nevertheless, in endocrine tissues virus detection was significantly more common in individuals with obesity. (2) Not all endocrine organs could be tested in all autopsy cases, thus limiting estimates of infection prevalence per tissue and concordance analyses. (3) The detected virus variants are those prevalent in the first phase of the pandemic, thus precluding conclusions with regard to virus variants that circulate at the present time.

In closing, the spread of SARS-CoV-2 to endocrine organs appears to be common in lethal cases of COVID-19. Not all endocrine organs are equally prone to viral infection, with the anterior pituitary and WAT representing the most common targets. Remarkably, the frequent finding of virus in endocrine organs reinforces the link of adiposity-based chronic disease with disease severity. In addition to the rapid decline of the humoral response after vaccination in subjects with high BMI values [41], the present findings highlight the importance of vaccination against SARS-CoV-2 for these individuals. Since post-COVID conditions remain a major issue in the current epidemic, a rigorous follow-up of endocrine function—especially of thyroid and pancreas—is advocated in subjects with obesity.

## Data Availability

Original data generated and analyzed during this study are included in this published article.
